# Clinical outcomes of cryptozoospermic patients undergoing surgical sperm retrieval

**DOI:** 10.3389/fruro.2023.1160122

**Published:** 2023-05-09

**Authors:** Raneen Sawaid Kaiyal, Rossella Cannarella, Shinnosuke Kuroda, Neel V. Parekh, Sarah C. Vij, Scott D. Lundy

**Affiliations:** Glickman Urological & Kidney Institute, Cleveland Clinic Foundation, Urology Department, Cleveland, OH, United States

**Keywords:** male infertility, cryptozoospermia, ICSI, testicular sperm, micro TESE

## Abstract

**Introduction:**

Cryptozoospermia is defined by the World Health Organization (WHO) as the presence of isolated sperm cell in the ejaculate only identified after an extended microscopic search or after being pelleted. Although the number of spermatozoa is usually sufficient for intracytoplasmic sperm injection (ICSI), ICSI fails due to poor sperm quality in some cases. Contention remains regarding whether testicular sperm offers any advantage in this unique situation. At our tertiary referral center, we will offer patients a surgical sperm retrieval via conventional or microdissection testicular sperm extraction (microTESE) for men with cryptozoospermia and failed ICSI, or where ejaculated specimens are immotile or insufficient for ICSI. In this study, we sought to describe our experience and evaluate the predictors of success in cryptozoospermic patients who had microTESE at our center.

**Methods:**

We retrospectively reviewed our electronic medical records for all patients with cryptozoospermia who underwent microTESE between 2007- 2021 for failed ICSI with ejaculated sperm or sperm quality deemed to be of insufficient quality for ICSI (e.g., nonmotile sperm). We evaluated demographics, preoperative lab results, pathology results, sperm retrieval rate (SRR) and ICSI outcomes.

**Results:**

28 cryptozoospermic patients were identified. These patients underwent 37 unique microTESE. 22 of these men had failed previous ICSI treatment with ejaculated sperm, while the other 6 patients had ejaculated sperm with non-suitable quality for ICSI. None had genetic abnormalities. Successful retrieval of motile sperm suitable for ICSI was achieved in in 30 micro TESE procedures (SRR: 81.0%).14 out of 28 patients (50%) who underwent embryo transfer had positive pregnancy result, and 12/28 patients (42.8%) had successful live birth. The most common pathological pattern was hypospermatogenesis found in 65.3% (17/26). Fibrosis pathology was significantly higher in the negative pregnancy group. There were no postoperative complications noted.

**Disscussion:**

The use of testicular sperm in cryptozoospermic men with failed prior ICSI using ejaculated sperm has a high rate of pregnancy and live birth. While still controversial, our results suggest that surgical sperm retrieval is a viable option for these men with minimal risk of complications.

## 1 Introduction

Cryptozoospermia is a severe form of oligozoospermia defined by the World Health Organization (WHO) as the presence of isolated sperm cells in the ejaculate only identified after an extended microscopic search or after being pelleted (1).

Literature suggests that cryptozoospermia accounts for 8.73% of male infertility cases (2), but this incidence likely varies considerably according to baseline patient demographics and comorbidities, andrology lab expertise and protocols, and suitability or access for assisted reproductive technology (3).

Cryptozoospermia is caused by multiple etiologies including genetic alterations (Klinefelter syndrome, Y chromosome abnormalities, monogenic disorders), hormonal abnormalities (hypogonadotrophic hypogonadism), anatomic/obstructive causes due to infections (orchitis), surgeries (trauma or cancer), or cancer treatments (chemotherapy, radiation) (2).

The optimal treatment in cases of cryptozoospermia is Intracytoplasmic Sperm Injection (ICSI), but the ideal sperm source (ejaculated sperm versus testicular sperm) remains incompletely characterized in all cases (4). Testicular sperm does offer the theoretical benefit of decreased DNA fragmentation due the avoidance of sperm genomic content degradation during epididymal transit (5), but the sequential washing and centrifugation necessary conversely reduces sperm quality and cryopreservation yields (6).

Multiple observational studies on this topic reported different and contradicted results.

The debate continues even with the publication of three meta-analysis on this topic.

The first, published in 2015, found no difference in fertilization and pregnancy rates when using either testicular or ejaculated sperm for ICSI (7). In 2018 two other studies were reported. The first favored fresh testicular sperm for good quality embryo rate, implantation rate and pregnancy rate (8), but the second study reported results withn no significant difference in miscarriage between testicular sperm and ejaculated sperm. Importantly, however, the live birth date per embryo transfer in testicular sperm group was higher (9).

Taking all this into account, there is no robust evidence to show that cryptozoospermic patients have worse outcomes with ejaculate compared to testicular sperm-ICSI, even in absence of high DNA fragmentation. However, evidence still remains that severe oligozoospermia including cryptozoospermia and sperm DNA damage impact the success rate of ICSI when ejaculated sperm are used.

There is a need for further research on the optimal approach in cryptozoospermic patients.

In this study, we sought to describe our experience and evaluate the predictors of success in cryptozoospermic patients who had microsurgical testicular extraction microTESE at our centre.

## 2 Materials and methods

Following institutional review board approval (IRB approval number 18-1471), we performed a retrospective single tertiary institution analysis of all patients undergoing microTESE from 2007-2021.

We included only cases of microTESE which the male partner had cryptozoospermia and the female partner had normal fertility workup to minimize confounding factors. Cryptozoospermic patients were diagnosed according to the fifth edition of the WHO laboratory manual for the examination and processing of human semen.

According to our protocol, cryptozoospermic patients who failed ICSI with ejaculated sperm or who had immotile or morphologically poor sperm were offered microTESE with testicular sperm.

All patients were evaluated by an andrology specialist, with a full workup including detailed medical history regarding previous pregnancies, length of infertility, tobacco use, and EMT (Empirical Medical Therapy). The work up also included a physical examination including the presence of varicocele, a hormonal profile of testosterone, FSH (Follicular Stimulating Hormone), and LH (Luteinizing Hormone), as well as two semen analyses. All patients had genetics tests for Y-microdeletion and aneuploidy. Sonography of the testis and genital tract was recommended in cases of inconclusive or suspicious clinical findings.

MicroTESE was performed according to the initial description by Schlegel (10). Under general anesthesia, the testis were delivered individually using a midline scrotal incision. microTESE was then performed via an equatorial tunica albuginea incision and careful microscopic excision of heterogenous or full tubules. Samples were examined by a trained embryologist, and if necessary, the procedure was carried out on the contralateral testis. Specimens for pathology were sent bilaterally unless pathology had already been characterized.

We evaluated demographics, clinical history and physical exam, preoperative lab results, pathology results, sperm retrieval rate (SRR), pregnancy and live birth rates between the ejaculated sperm and testicular sperm. Our primary outcome of interest was clinical pregnancy.

Descriptive and comparative statistics were tabulated to identify patient demographics, clinical factors, pathology diagnosis in each testis and sperm retrieval rate.

Continuous variables were reported as median and confidence interval. The Wilcoxon rank-sum test was used to compare the two independent samples of patients with positive or negative pregnancy results. Discrete variables were reported as percentages, and comparison between patients with positive pregnancy result and those with negative ones was evaluated with the Fisher’s exact test. Threshold for statistical significance was p ≤ 0.05. Statistical analyses were performed using Medcalc®. Version 20.118 (MedCalc Software Ltd, Ostend, Belgium).

## 3 Results

28 cryptozoospermic patients were identified and underwent a total of 37 unique microTESE operations with pathology specimens sent in 26 of these operations. 22 of these patients had failed previous ICSI treatment with ejaculated sperm, while the other 6 patients had poor quality ejaculated sperm not suitable for ICSI. 14 operations were scheduled as frozen microTESE (sperm is retrieved ahead of egg retrieval with subsequent cryopreservation) and 23 were fresh microTESE (same day as egg retrieval).

Overall median age was 35.0 (29.5-38.4), median serum total testosterone level was 306 ng/dL (235-479), and median serum FSH level was 11.2 mIU/mL (5.8-21.8). Median length of infertility at time of microTESE was 36 months (18-60).

Motile sperm suitable for ICSI was retrieved in 82.1% of overall patients and 64.2% (9/14) of those who didn’t achieve pregnancy. The overall pregnancy rate was 50% (14 out of 28) and 12 patients (42.8%) had successful live birth.

Demographics and outcomes between the group of patients who achieved pregnancy using testicular sperm and those who did not are listed in **Table 1**.

**Table 1:** Patients characteristics and demographics.

	Positive Pregnancy (N=14)	Negative Pregnancy (N=14)	P value
Age, y	34.8 (28.9-42.9)	35.1(25.2-38.5)	0.718
Length of infertility*	42.0(23.3-121.2)	36.0(12.0-60.0)	0.158
Primary Infertility, % (n)	85.71 (12/14)	100(14/14)	0.777
Serum testosterone, ng/dL*	396.0(262.4-429.7)	393(402-261.5)	0.384
Serum FSH^1^, mIU/mL*	11.1(5.8-16.7)	12.8(4.0-25.9)	0.509
Serum LH^2^, mIU/mL*	7.3(6.0-8.9)	9.2(4.5-12.2)	0.222
Testicular volume, mL*	12(9.0-16.00)	11.0(7.0-15.0)	0.373
Tobacco use, % (n)^**^	64.2 (9/14)	28.5(4/14)	0.248
Clinical varicocele, % (n)^**^	42.8 (6/14)	35.7 (5/14)	0.490
Surgery while on EMT^3%^, (n)^**^	28.4 (4/14)	28.4 (4/14)	1
Klinefelter Syndrome % (n)**	0(0/14)	0(0/14)	1
Y Chromosome -microdeletion% (n)**	0 (0/14)	0 (0/14)	1
Live birth rate, %(n)^**^	85.7 (12/14)	0 (0/14)	
Sertoli Cell only %(n)^**^	11.5 (3/26)	34.6 (9/26)	0.116
Fibrosis%(n)**	0 (0/26)	7.6 (2/26)	0.028
Maturation Arrest %(n)**	23 (6/26)	23.0 (6/26)	1
Leydig Cell Hyperplasia %(n)**	0 (0/26)	0 (0/26)	1
Hypo spermatogenesis %(n)**	65.3 (17/26)	34.6(6/26)	0.053
**Frozen sperm**	35.7 (5/14)	42.8 (6/14)	1

^1^FSH: Follicular Stimulating Hormone, ^2^LH: Luteinizing Hormone, ^3^EMT, Empirical Medical Therapy.

*Values are Median and Confidence Interval, Fisher’s exact test.

**Values are Percentage and Wilcoxon rank-sum test.

Statistically significant (p < 0.05).

No significant different was observed between the two groups regarding age, fertility type and length of infertility as well as laboratory results, smoking and presence of varicocele. None had genetic abnormalities.

The most common pathological pattern was hypospermatogenesis found in 65.3% (17/26).

Fibrosis pathology was significantly higher in the negative pregnancy group (0% vs 7.6%, p=0.028).

There were no no male surgical complications noted.

## 4 Discussion

The advent of ICSI technique in 1992 has revolutionized the treatment of most male infertility conditions including azoospermia. In case of cryptozoospermia, it remains unclear whether testicular sperm offers any meaningful benefit regarding clinical pregnancy rate over ejaculated sperm.

Ejaculated sperm is easy to collect, less expensive and lack any complications while testicular sperm collection is much more complicated, invasive, expensive and could cause complications related to surgery and general anesthesia. Ejaculated sperm in cryptozoospermic men, however, likely contains additional risks of DNA damage occurring at the post testicular (e.g. epididymal and vasal) level (11). Sperm with high index of DNA fragmentation has been associated with compromised clinical outcomes (12). Additionally, centrifugation (as is often necessary in cryptozoospermic men) leads to both nitrous oxide (NO) and reactive oxygen species (ROS) production with detrimental effects on both sperm motility and viability (6).

Despite these facts, studies addressing the source of sperm used in cryptozoospermic patients reported contradicted results. Some cohort studies showed that ICSI outcomes are not compromised by the origin of sperm cells (13). Other studies have reported that ICSI outcomes are superior using ejaculated sperm (14). Still other studies have reported that extremely low numbers of ejaculated sperm are related to compromised fertilization and pregnancy rates (15), and testicular sperm may be associated with higher implantation, pregnancy rate, and live birth (4, 16, 17). This contradiction could be attributed to that fact that different studies reported different primary outcome using different patient populations. When the primary outcome being analyzed is the fertilization rate, most published results have shown no difference between ejaculated and testicular sperm (18), and some have even demonstrated beneficial different toward ejaculated sperm.

However, when other parameters such as implantation, miscarriages, and live birth were included, almost all studies reported that results were in favor of testicular sperm as highly fragmented sperm can fertilize an egg but has lower implantation potential and hence higher miscarriage rates and lower pregnancies (19). The only exception for this was a study published by Ubaldi et al (14). in 1999 who reported that using testicular sperm for ICSI in non-obstructive azoospermia resulted in significantly lower implantation rate than that in the matched ejaculated sperm group, however the matched controlled group in his study was not cryptozoospermic patients, rather it included patients with severe oligospermia (sperm concentration <5x10^6^/ml) which is a different group with better outcomes.

In our study we aimed to shed some light on this controversial topic by summarizing our treatment experience with cryptozoospermic patients and looking for differences and predictors of success. In the lack of clear advantage and recommendations, we initially offer our patients ICSI cycle using ejaculated sperm, in cases of ICSI failure or in cases when we don’t have suitable ejaculated sperm for ICSI (immotile or severely abnormal morphology) we proceed with microTESE and use testicular sperm.

Our results show that this approach is highly productive with clinical pregnancy rate of 50% and live birth rate of 42.8% with nomale surgical complications. These numbers compare even more favorably to alternatives when considering that this population had largely already failed ICSI previously.

## 5 Conclusion

The use of testicular sperm in cryptozoospermic men with failed prior ICSI using ejaculated sperm has a high rate of pregnancy and live birth. Whether these patients would have been just as successful with ejaculated sperm is not known, but our results suggest that surgical sperm retrieval is a viable option for these men with minimal risk of complications and should be offered to cryptozoospermic men with failed ICSI or severely compromised specimen quality.

## Data availability statement

The raw data supporting the conclusions of this article will be made available by the authors, without undue reservation.

## Ethics statement

The studies involving human participants were reviewed and approved by Institutional review board, Cleveland Clinic Foundation, Cleveland, OHIO, US. The ethics committee waived the requirement of written informed consent for participation.

## Author contributions

RS and SL designed the study. RS collected data, perfume the statistical analysis, and drafted the manuscript. SL reviewed and revised the manuscript. All authors contributed to the article and approved the submitted version. 

## References

[1] World Health Organization. WHO laboratory manual for the examination and processing of human semen sixth edition. world health organization, Edition, V. (Geneva, Switzerland: WHO Press, World Health Organization) (2021).

[2] Karabulut S Keskin İ Kutlu P Delikara N Atvar Ö Öztürk M. Male Infertility, azoozpermia and cryptozoospermia incidence among three infertility clinics in Turkey. Turkish J Urol (2018) 44(2):109–35. doi: 10.5152/tud.2018.59196 PMC5832370 29511578

[3] Sermondade N Faure C Fezeu L Shayeb AG Bonde JP Jensen TK. BMI in relation to sperm count: an updated systematic review and collaborative meta-analysis association between BMI and sperm count abnormality sensitivity analyses assessment of publication bias † discussion. Hum Reprod Update (2013) 19(3):221–31. doi: 10.1093/humupd/dms050 PMC3621293 23242914

[4] Ben-ami I Raziel A Strassburger D Komarovsky D. Intracytoplasmic sperm injection outcome of ejaculated versus extracted testicular spermatozoa in cryptozoospermic men. Fertility Sterility (2013) 99(7):1867–715. doi: 10.1016/j.fertnstert.2013.02.025 23490166

[5] Suganuma R Yanagimachi R Meistrich. ML. Decline in fertility of mouse sperm with abnormal chromatin during epididymal passage as revealed by ICSI. Hum Reprod (2005) 20(11):3101–85. doi: 10.1093/humrep/dei169 16037114

[6] Lampiao F Strijdom H Du Plessis SS. Effects of sperm processing techniques involving centrifugation on nitric oxide, reactive oxygen species generation and sperm function. Open Andrology J (2010) 2:1–5. doi: 10.2174/1876827X01002010001

[7] Abhyankar N Kathrins M Niederberger C. Use of testicular versus ejaculated sperm for intracytoplasmic sperm injection among men with Cryptozoospermia : a meta-analysis. Fertility Sterility (2015) 105(6):1469–14755.e1. doi: 10.1016/j.fertnstert.2016.02.013 26930617

[8] Kang Y-n Hsiao Y-w Chen C-y Wu. C-c. Testicular sperm is superior to ejaculated sperm for ICSI in Cryptozoospermia : an update systematic review and meta- analysis. Sci Rep (2018) 8(1):7874. doi: 10.1038/s41598-018-26280-0 29777145 PMC5959851

[9] Ku FY Wu CC Hsiao YW Kang YN. Association of sperm source with miscarriage and take-home baby after ICSI in cryptozoospermia: a meta-analysis of testicular and ejaculated sperm. Andrology (2018) 6(6):882–9. doi: 10.1111/andr.12546 30207082

[10] Schlegel PN. Testicular sperm extraction: microdissection improves sperm yield with minimal tissue excision. Hum Reprod (1999) 14(1):131–5. doi: 10.1093/humrep/14.1.131 10374109

[11] Greco E Scarselli F Iacobelli M Rienzi L Ubaldi F Ferrero S. Efficient treatment of infertility due to sperm DNA damage by ICSI with testicular spermatozoa (Accessed January 23, 2023). 10.1093/humrep/deh590 15539441

[12] Zhu C Chen F Zhang S She H Ju Y Wen X. Influence of sperm DNA fragmentation on the clinical outcome of *in vitro* fertilization-embryo transfer (IVF-ET). Front Endocrinol (2022) 13(July):1607. doi: 10.3389/FENDO.2022.945242/BIBTEX PMC9329669 35909570

[13] Bukulmez O Yucel A Yarali H Bildirici I Gurgan T. The origin of spermatozoa does not affect intracytoplasmic sperm injection outcome. Eur J Obstet Gynecol Reprod Biol (2001) 94(2):250–5. doi: 10.1016/S0301-2115(00)00347-X 11165734

[14] Ubaldi F Nagy ZP Rienzi L Tesarik J Anniballo R Franco G. Reproductive capacity of spermatozoa from men with testicular failure. Hum Reprod (1999) 14(11):2796–28005. doi: 10.1093/humrep/14.11.2796 10548625

[15] Strassburger D Friedler S Raziel A Schachter M Kasterstein E Ron-El R. Very low sperm count affects the result of intracytoplasmic sperm injection. J Assisted Reprod Genet (2000) 17(8):431–36. doi: 10.1023/A:1009413201849 PMC3455570 11062853

[16] Hauser RON Bibi GUY Yogev L Carmon A Azem F Botchan A. Virtual azoospermia and cryptozoospermia — Fresh/Frozen. J Androl (2011) 32(5):484–90. doi: 10.2164/jandrol.110.011353 21164144

[17] Cui X Ding P Gao G Zhang. Y. Comparison of the clinical outcomes of intracytoplasmic sperm injection testicular biopsy and from ejaculate. Urology (2016) 102:106–. doi: 10.1016/j.urology.2016.08.071 27894976

[18] Amirjannati N Heidari-Vala H Akhondi MA Hosseini Jadda SH Kamali K Sadeghi MR. Comparison of intracytoplasmic sperm injection outcomes between spermatozoa retrieved from testicular biopsy and from ejaculation in cryptozoospermic men. Andrologia (2012) 44(Supp 1):704–9. doi: 10.1111/j.1439-0272.2011.01253.x 22077321

[19] Sedó CA Bilinski M Lorenzi D Uriondo H Noblía F Longobucco V. Effect of sperm DNA fragmentation on embryo development: clinical and biological aspects. Jornal Brasileiro Reproducao Assistida (2017) 21(4):343–505. doi: 10.5935/1518-0557.20170061 PMC5714603 29116706

